# Case report on postoperative coagulation abnormalities

**DOI:** 10.1097/MD.0000000000036618

**Published:** 2024-01-05

**Authors:** Hongjing Zhou, Yanyan Tang, Yongtian Zhang, Yejing Zhu, Shasha Dong

**Affiliations:** a Jining No. 1 People’s Hospital, Jining, Shandong, China; b Shandong Daizhuang Hospital, Jining, Shandong, China.

**Keywords:** coagulation abnormalities, craniocerebral surgery, intracranial aneurysm, lupus anticoagulant, trauma-induced coagulopathy

## Abstract

**Rationale:**

Intracranial aneurysm (IA) is defined as a localized dilation of cerebral arteries. With the continuous development of modern medical technology, surgery is still one of the main treatment methods. Although there are various postoperative complications, abnormal coagulation function is rare, especially those caused by lupus antibodies after surgery. The patient not only experienced postoperative abnormalities in coagulation function, but also discovered the presence of lupus anticoagulants in their body. Is the patient suffering from coagulation dysfunction caused by lupus anticoagulants, how is lupus anticoagulant produced, and what’s special about treatment. With these questions in mind, we reviewed the entire treatment process of the patient.

**Patient concerns:**

A 69-year-old woman presented with “headache and dizziness with neck pain” and was eventually diagnosed with IA hemorrhage. The patient underwent craniotomy under general anesthesia, and provided targeted support and treatment. Postoperative symptoms such as coma and intermittent fever occurred, and coagulation indicators were generally normal. After symptomatic support treatment, such as anti-infection treatment, the patient’s temperature was gradually controlled. However, the abnormal clotting index and the efficacy of symptomatic therapeutic support, such as supplementation with coagulation factors, were not good. After further examination, the lupus anticoagulant was found, which provided us with a new treatment idea.

**Diagnoses:**

Coagulation disorders, postoperative IA, hypertension grade 3 (extremely high risk), coronary atherosclerotic atheropathy, and type 2 diabetes.

**Interventions:**

The patient developed abnormal coagulation function after craniotomy, and symptomatic support treatment with coagulation factor supplementation and plasma infusion was ineffective. Finally, the lupus anticoagulant was found after a series of relevant examinations. After timely adjustment of the treatment plan, the patient’s coagulation indices gradually improved.

**Outcomes:**

In this report, we present the case of a patient with abnormal coagulation function caused by the lupus anticoagulant after IA surgery.

**Lessons:**

The coagulation function of the patient was abnormal after craniocerebral operation. After coagulation factor supplementation, the coagulation index of the patient was still not well improved. After further examination, the lupus anticoagulant was found. The treatment plan was actively adjusted, and the patient’s condition gradually improved. Early recognition can allow doctors to provide appropriate therapy to patients.

## 1. Introduction

Intracranial aneurysms (IAs) are life-threatening cerebrovascular pathologies with an incidence and prevalence of 1.6% and 3.2%, respectively, in the adult population. The peak age is between 55 and 64 years old. IAs are thought to result from an abnormal thickening of the artery wall at sites where hemodynamic stress is high.^[1]^ Hypertension and arteriosclerosis can cause damage to the inner wall of intracranial arteries. Muscle cells with smooth vascular walls have poor tolerance to ischemia and hypoxia and are prone to aneurysm formation, which is one of the reasons for aneurysm enlargement and rupture. Unruptured IAs are generally silent but become symptomatic when they rupture and cause subarachnoid hemorrhage, the mortality rates are approximately 30% to 40%, and severe neurological dysfunction and disability affect a large proportion of subarachnoid hemorrhage survivors.^[2–4]^

Coagulation dysfunction is a bleeding disease caused by deficiency or abnormal function of coagulation factors. There are 2 categories: hereditary and acquired. Acquired coagulation dysfunction is common, and patients often lack a variety of coagulation factors. Multiple diseases, such as liver disease and disseminated intravascular coagulation, trauma, and the use of anticoagulants, can lead to coagulopathy. Trauma-induced coagulopathy is more common. Trauma-induced coagulopathy is a disorder of coagulation function caused by the activation of coagulation, anticoagulation, and fibrinolysis processes due to bleeding, tissue destruction, and other reasons in severe trauma. It is a multiple coagulation disorder. However, the occurrence of coagulation dysfunction caused by lupus anticoagulants after trauma is extremely rare, and the prognosis varies.^[5]^ In this case, we found the lupus anticoagulant in a patient with postoperative coagulation dysfunction of an IA. This patient did not have abnormal coagulation function indicators before surgery. To the best of our knowledge, this case has not been reported.

## 2. Case presentation

**Figure 1.**
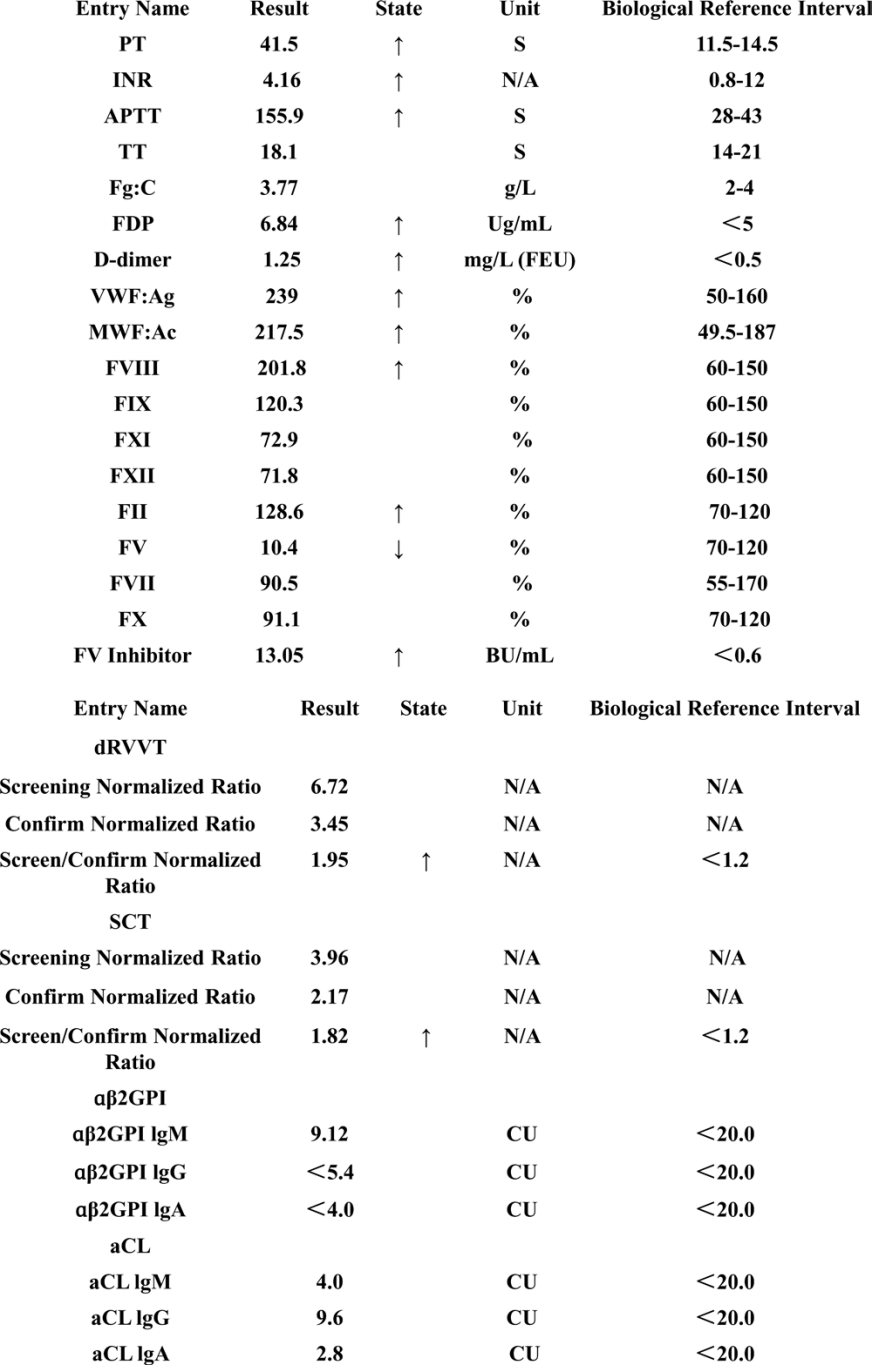
Determination of plasma prothrombin time and activated partial thromboplastin time extension package.

A 69-year-old woman with a history of hypertension grade 3 (extremely high risk), coronary heart disease, and diabetes was hospitalized with headache, dizziness, and neck pain. After the examination, she was diagnosed with subarachnoid hemorrhage and IA hemorrhage. After communicating with the family members, further action, including interventional embolization of the aneurysm, was suggested. Routine blood examination, coagulation function, and 4 other items were normal before the operation. Craniotomy (intracranial aneurysm clipping and artificial dura mater) under general anesthesia was performed, with approximately 200 mL of blood loss and 100 mL of autologous blood that was transfused during the operation; the total infusion volume was 1500 mL. After the operation, the patient was in a coma and had intermittent fever, with the highest body temperature being 39ºC. Inflammatory indicators, such as white blood cells (14.49 × 10^9^/L), neutrophils (12.32 × 10^9^/L), C-reactive protein (139.34 mg/L), and procalcitonin (0.14 ng/mL), were high, and coagulation indicators were abnormal. After symptomatic support treatment, such as anti-inflammatory therapy, fluid infusion, intracranial pressure reduction, and epilepsy prevention, the patient’s consciousness gradually became clear, the body temperature returned to normal, and her inflammatory indicators, such as white blood cells (9.23 × 10^9^/L), neutrophils (5.77 × 10^9^/L), C-reactive protein (76.19 mg/L), and procalcitonin (0.08 ng/mL), returned to normal. However, after repeated examination, coagulation was abnormal, so we considered coagulation dysfunction. She received a vitamin K1 intravenous drip, coagulation factor supplementation, a plasma infusion and other symptomatic support treatment, and the effect was not good. After all the coagulation factors returned to normal, the lupus anticoagulant was found, coagulation Factor V was slightly deficient, and there was a high titer of factor V (one of blood clotting factors) inhibitor, which did not rule out factor inhibition due to the interference of the lupus anticoagulant (Fig. 1). The antinuclear antibody spectrum and anticardiolipin antibody are generally normal. At present, the patient is positive for the lupus anticoagulant, and the efficacy of transfusion of coagulation factors and plasma is not good. The treatment plan was adjusted and glucocorticoids were added. After treatment, the patient’s blood coagulation function was normal. It is suggested to reexamine patients with the lupus anticoagulant after 3 months to exclude phospholipid antibody syndrome.

## 3. Discussion

Coagulation dysfunction after craniocerebral surgery is a complication of neurosurgery emergency that has a high incidence of 56% to 70%.^[6]^ Coagulation dysfunction after craniocerebral injury mainly includes posttraumatic hypercoagulability and secondary fibrinolytic hyperactivity, mainly including the C-protein activation hypothesis, endothelial activation, platelet activation, fibrinolytic hyperactivity, and the microparticle hypothesis, which greatly increases the risk of rebleeding and death after craniocerebral injury and seriously affects the prognosis of patients. First, craniotomy can lead to hypercoagulability. Because of the characteristics of brain tissue, it is more prone to cause blood hypercoagulability than other tissues when it is injured. Brain tissue contains rich tissue factors.^[7]^ At the same time, brain tissue also has the richest blood circulation in the human body. Brain tissue and vascular endothelial cells are damaged during surgery. The blood-brain barrier is damaged, and a large number of tissue factors are released into the blood, thus stimulating the exogenous coagulation system. This is the most abundant source of thrombus-activating enzymes in the body and makes the coagulation function of the local circulation in the injured area more significant than that of the systemic circulation, and the incidence of these coagulation diseases is related to the severity or degree of brain tissue injury, sometimes as a result of surgery.^[8]^ Second, a craniocerebral injury can activate the blood coagulation system and reduce platelets and fibrinogen. Craniotomy can stimulate and influence the coagulation system with multiple factors, which can further aggravate the abnormalities of coagulation and fibrinolysis. Some patients may have disseminated intravascular coagulation, which has a high mortality and disability rate.^[9]^ Finally, hypothermia, blood transfusion products, antibiotic use, and other factors can also affect coagulation function to a certain extent. Hypothermia can damage coagulation factors, reduce thrombin activity, reduce the number and function of platelets, release heparin-like substances, reduce blood volume, increase blood viscosity and plasma concentration, and finally cause a coagulation disorder. During surgical treatment of IAs, the change in blood volume and excessive input of exogenous blood products, crystal liquid, and colloid liquid are the causes of blood coagulation disorder. The use of antibacterial agents will further cause adverse reactions in the blood system. For example, cephalosporins can cause blood coagulation dysfunction and block vitamin K, which is a necessary cofactor of liver microsomal spindle enzyme and is a necessary condition for the γ-carboxylation of glutamate in prothrombin precursors. Vitamin K deficiency can reduce the synthesis of prothrombin and reduce the content of vitamin K-dependent coagulation factors II, VII, IX, X, etc, thus causing coagulation disorders.^[10,11]^

The influence of the above factors on the patient’s blood coagulation function after surgery can be gradually recovered after symptomatic support treatment, such as coagulation factor supplementation and blood product transfusion. However, the patient’s blood coagulation function has not been improved very well, which not only makes us think about whether the patient has other special diseases that interfere with the blood coagulation process, but the patient has no relevant medical history. After discussion, we further improved the relevant immunological indicators and coagulation factors to evaluate whether patients had immunologically recessive diseases and lupus anticoagulants. However, the patient did not have any autoimmune disease in the past, nor did he take psychotropic drugs for a long time. The patient’s relevant immunological indicators were normal. Why did new lupus anticoagulants appear after surgery? This helped us to brainstorm. Since blood hypercoagulability, hypothermia, exogenous transfusion of blood products, and the application of antibiotics can affect the blood coagulation function after surgery, does this mean that these exogenous stimuli trigger the body’s defense system, interfere with the body’s endocrine and immune systems, trigger their own over reaction, and further trigger the production of new antibodies? Was the lupus anticoagulant in the body before, but did not show? After this craniotomy, a series of factors had positive expressions and interfered with blood coagulation. Moreover, the patient’s prothrombin time and activated partial thromboplastin time extended packages were not reexamined after 12 weeks of treatment, and it was not clear whether lupus anticoagulants persisted or appeared temporarily. For patients with hypertension, diabetes, and coronary heart disease in the past, whether these factors will also affect the positive expression of lupus anticoagulants needs further research and discussion. In summary, the stress of surgery, the patient’s own immune status, the involvement of exogenous factors, and the patient’s comorbidities, all various factors that interfere with the internal environment may be the reasons for the production of lupus anticoagulants. After treatment, the patient was not well followed up, resulting in a lack of clear understanding of the specific cause of lupus anticoagulants. Although we did not further analyze the specific causes of the lupus anticoagulant this time, it guided us to continue to treat coagulation dysfunction after craniocerebral surgery. Once the patient’s coagulation has not been effectively improved after blood products and coagulation factors have been transfused, it is necessary to consider whether there are other factors affecting it. The lupus anticoagulant is one of the influencing factors, and its early recognition can allow doctors to provide appropriate and timely therapy to patients.

## Acknowledgments

We are very grateful to the teachers of Jining No. 1 People’s Hospital and Jining Medical College for their support and Mrs. Wei’s cooperation.

## Author contributions

**Data curation:** Hongjing Zhou, Yongtian Zhang.

**Project administration:** Shasha Dong, Yejing Zhu.

**Resources:** Yanyan Tang, Yongtian Zhang.

**Writing – original draft:** Yanyan Tang, Yejing Zhu.

**Writing – review & editing:** Hongjing Zhou, Shasha Dong.

## References

[1] Juvela S. Prevalence of and risk factors for intracranial aneurysms. Lancet Neurol. 2011;10:595–7. 21641283 10.1016/S1474-4422(11)70125-9

[2] Frösen J Tulamo R Paetau A. Saccular intracranial aneurysm: pathology and mechanisms. Acta Neuropathol. 2012;123:773–86. 22249619 10.1007/s00401-011-0939-3

[3] Petridis AK Kamp MA Cornelius JF. Aneurysmal subarachnoid hemorrhage. Dtsch Arztebl Int. 2017;114:226–36. 28434443 10.3238/arztebl.2017.0226 PMC5624452

[4] van Gijn J Kerr RS Rinkel GJ. Subarachnoid haemorrhage. Lancet. 2007;369:306–18. 17258671 10.1016/S0140-6736(07)60153-6

[5] Moore EE Moore HB Kornblith LZ. Trauma-induced coagulopathy. Nat Rev Dis Primers. 2021;7:30. 33927200 10.1038/s41572-021-00264-3 PMC9107773

[6] Kutcher ME Ferguson AR Cohen MJ. A principal component analysis of coagulation after trauma. J Trauma Acute Care Surg. 2013;74:1223–9; discussion 1229–30. 23609271 10.1097/TA.0b013e31828b7fa1 PMC3638007

[7] Maegele M Schöchl H Menovsky T. Coagulopathy and haemorrhagic progression in traumatic brain injury: advances in mechanisms, diagnosis, and management. Lancet Neurol. 2017;16:630–47. 28721927 10.1016/S1474-4422(17)30197-7

[8] Green J Doughty L Kaplan SS. The tissue factor and plasminogen activator inhibitor type-1 response in pediatric sepsis-induced multiple organ failure. Thromb Haemost. 2002;87:218–23. 11858480

[9] van der Sande JJ Veltkamp JJ Boekhout-Mussert RJ. Head injury and coagulation disorders. J Neurosurg. 1978;49:357–65. 681997 10.3171/jns.1978.49.3.0357

[10] Jacoby RC Owings JT Holmes J. Platelet activation and function after trauma. J Trauma. 2001;51:639–47. 11586152 10.1097/00005373-200110000-00003

[11] Kunio NR Differding JA Watson KM. Thrombelastography-identified coagulopathy is associated with increased morbidity and mortality after traumatic brain injury. Am J Surg. 2012;203:584–8. 22425448 10.1016/j.amjsurg.2011.12.011

